# The effects of self-selected light-dark cycles and social constraints on human sleep and circadian timing: a modeling approach

**DOI:** 10.1038/srep45158

**Published:** 2017-03-27

**Authors:** Anne C. Skeldon, Andrew J. K. Phillips, Derk-Jan Dijk

**Affiliations:** 1 University of Surrey, Department of Mathematics, Guildford, GU2 7XH, UK; 2 Harvard Medical School, Division of Sleep and Circadian Disorders, Brigham and Women’s Hospital, USA; 3 University of Surrey, Surrey Sleep Research Centre, Guildford, GU2 7XP, UK

## Abstract

Why do we go to sleep late and struggle to wake up on time? Historically, light-dark cycles were dictated by the solar day, but now humans can extend light exposure by switching on artificial lights. We use a mathematical model incorporating effects of light, circadian rhythmicity and sleep homeostasis to provide a quantitative theoretical framework to understand effects of modern patterns of light consumption on the human circadian system. The model shows that without artificial light humans wakeup at dawn. Artificial light delays circadian rhythmicity and preferred sleep timing and compromises synchronisation to the solar day when wake-times are not enforced. When wake-times are enforced by social constraints, such as work or school, artificial light induces a mismatch between sleep timing and circadian rhythmicity (‘social jet-lag’). The model implies that developmental changes in sleep homeostasis and circadian amplitude make adolescents particularly sensitive to effects of light consumption. The model predicts that ameliorating social jet-lag is more effectively achieved by reducing evening light consumption than by delaying social constraints, particularly in individuals with slow circadian clocks or when imposed wake-times occur after sunrise. These theory-informed predictions may aid design of interventions to prevent and treat circadian rhythm-sleep disorders and social jet-lag.

Circadian clocks evolved as an adaptive response to daily environmental cycles, such as the light-dark cycle, associated with the Earth’s rotation. Circadian rhythms are present at the molecular level in nearly all cells of the body and brain^1^. In mammals, peripheral rhythms are orchestrated by a central circadian pacemaker located in the suprachiasmatic nucleus (SCN) of the hypothalamus and are also influenced by behaviors such as feeding. This circadian system assures that biochemical, physiological, and behavioral processes, including the timing of sleep and wake, occur at a species-specific appropriate time of day^2^.

The intrinsic period of the circadian pacemaker deviates from 24 hours and is estimated to average 24 h 9 min ± 12 (SD) min in humans^3^. The circadian pacemaker is entrained to 24 hours by ‘zeitgebers’ (literally, ‘time givers’). In humans, the light-dark cycle is a powerful zeitgeber and exerts its influence through a specialized photoreceptive and phototransductive pathway that impinges on the molecular component of the circadian clock in the SCN^4^. In the absence of an externally-imposed 24-hour light-dark cycle — for example, in completely blind individuals or when humans self-select their light-dark cycles — sleep-wake and other rhythms remain, but are no longer synchronized to the 24-hour cycle^5^,^6^,^7^.

Thus, the standard exposition of the circadian system is that it is an endogenous oscillation that is entrained to 24 hours by the light-dark cycle. Predictions that follow from this are that the phase of entrainment (i.e. how the rhythm is aligned relative to the light-dark cycle) should depend on both the intrinsic period of the circadian clock and the strength of the entraining signal. Both have been repeatedly confirmed in laboratory experiments^6^,^8^. Light is able to entrain the circadian clock because it either accelerates (advances) or retards (delays) the clock’s rhythm, depending when in the circadian cycle it is received^9^. The efficacy of a light stimulus also depends on its duration, irradiance and spectral composition^4^. The sensitivity of the human circadian pacemaker to light has a highly nonlinear dose response so that light levels similar to those observed in living rooms in the evening have been shown to affect circadian physiology and sleep timing^10^,^11^. Mathematical models of the effects of light on the human circadian clock have been developed to accurately describe these and other properties of the system^12^,^13^.

The timing of the human sleep-wake cycle is regulated not only by the endogenous circadian clock, which generates a circadian rhythm in sleep-wake propensity, but also by a relaxation oscillator called the sleep homeostat^14^. This second oscillator describes the physiological need for sleep, known as homeostatic sleep pressure, which increases during wakefulness and dissipates during sleep. In humans, the phase relationship between the two oscillators facilitates the consolidation of the sleep-wake cycle and the smooth transition between sleep and wakefulness. During the day, circadian wake propensity increases and thereby opposes the wake-dependent increase in sleep propensity; during the night, circadian sleep propensity increases and thereby facilitates the continuation of sleep despite the sleep-dependent dissipation of sleep pressure^15^. Typical profiles for the circadian wake propensity rhythm and the homeostatic sleep pressure are shown in Fig. 1. The interaction between the two oscillators has been confirmed in many experiments^16^,^17^,^18^ and is formalized in the current standard model of sleep regulation^19^.

The advent of electric light has given us much greater control over the light-dark cycle to which we are exposed and choice over when we sleep by delaying bed-time. Wake-time is often triggered by alarm clocks set to comply with social demands, such as work schedules and school start-times. In addition, humans in industrialized societies spend much more time inside, thereby reducing day-time light exposure by an order of magnitude relative to pre-industrialized societies^20^,^21^,^22^. This modern lifestyle is assumed to be associated with a mismatch between our sleep schedules and endogenous circadian rhythmicity. During work days we wake up early, out of phase with our circadian wake propensity rhythm, and obtain insufficient sleep. During ‘free days’ we sleep later, closer to our natural circadian wake propensity rhythm, and also longer, to pay off accrued sleep debt. This weekly pattern of sleeping in and out of phase with circadian rhythmicity has been coined ‘social jet-lag’^23^, drawing parallels between this weekly pattern of misalignment and the misalignment induced by rapid travel across time zones (jet-lag). A schematic diagram contrasting light profiles, the circadian wake propensity and sleep homeostat and resultant sleep timing is shown in Fig. 1 for two light profiles, one representative of pre-industrialized societies and one that is typical of modern life.

Insufficient sleep, and a temporal mismatch between sleep and circadian rhythmicity, are associated with adverse mental and physical health outcomes^24^. Proposed remedies for social jet-lag and associated sleep loss are individualized work schedules and later school start-times^25^. While there have been some empirical case studies, there are currently no standard theory-based quantitative evaluations of the effects of such interventions on circadian rhythmicity and sleep timing. Furthermore, the theoretical impact of self-selection of light exposure on entrainment of the human circadian oscillator and sleep timing has not been explored. Understanding these phenomena is increasingly important as the light from modern lamps, such as LEDs, typically contains a greater percentage of blue light, making them more biologically effective, and the use of devices that emit artificial light (e.g., smartphones) becomes more pervasive^4^,^26^,^27^.

Here, we use a mathematical model^28^ that incorporates the standard model for the effects of light on circadian rhythmicity and the standard model for the circadian and homeostatic regulation of sleep timing to investigate the effects of self-selected light exposure and social schedules on sleep and circadian timing. This model has been shown to describe changes in sleep timing and duration across the lifespan for a modern industrialized country^29^. We demonstrate that the model also agrees well with measurements of sleep timing and duration in pre-industrialized societies. The fact that the model quantitatively replicates observed features of sleep timing in societies that experience different extremes of the light environment suggests that it is a good representation of the interaction of light with circadian rhythmicity and sleep/wake timing. We therefore present results of a systematic investigation of the impact of natural and self-selected artificial light and social schedules on circadian rhythmicity, sleep timing, sleep duration, and social jet-lag. In addition, we explore (i) the relative effectiveness of changing the timing of social constraints versus changing light consumption behavior on circadian rhythmicity, social jet-lag, sleep duration, and day-time sleepiness; and (ii) the extent to which changes in sleep timing across the lifespan are modified by light consumption behaviors. By understanding the underlying theoretical structure of the model, we provide a unifying framework for understanding how light consumption and social constraints interact to determine sleep timing.

## Results

### In the absence of artificial light, we wake at dawn

**Figure 1:**
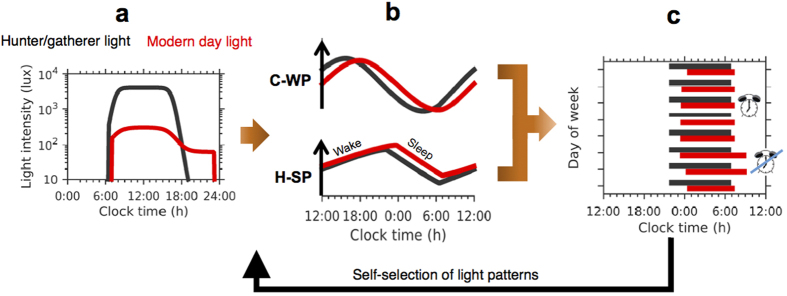
Schematic diagram of the impact of ancestral versus modern day light exposure and social constraints on sleep patterns. (**a**) Light input into the circadian pacemaker representative of hunter/gatherers in black and humans in industrialised societies in red. In both conditions the light input depicted is gated by the sleep-wake cycle, i.e. light input occurs during wake but not during sleep. By switching on electric light modern humans can extend the light period. (**b**) Core processes that govern wake/sleep timing are the circadian wake/sleep propensity rhythm and the sleep homeostat. Under normal conditions, circadian wake propensity (C-WP) is high during the day (wakefulness) and low during the night (sleep). Homeostatic sleep propensity (H-SP) increases during wakefulness and dissipates during sleep. The C-WP and the H-WP are shown in the absence/presence of evening light in black/red respectively. (**c**) Evening light exposure delays the timing of sleep onset and the circadian rhythm, but social constraints, indicated by the alarm clock, force us to wake up early. In the absence of social contraints, indicated by the crossed out alarm clock, we wake up later, in phase with the delayed circadian clock. Overall the self-selection of light leads to a delayed and irregular sleep pattern. Sleep timing is shown in the absence/presence of evening light in black/red respectively.

The mathematical model takes light as an input and produces time courses for the circadian wake propensity, the sleep homeostat and sleep-wake timing. Full details of the model are given in the Methods section and in the Supplementary material, but typical outputs are shown in Fig. 1.

**Figure 2:**
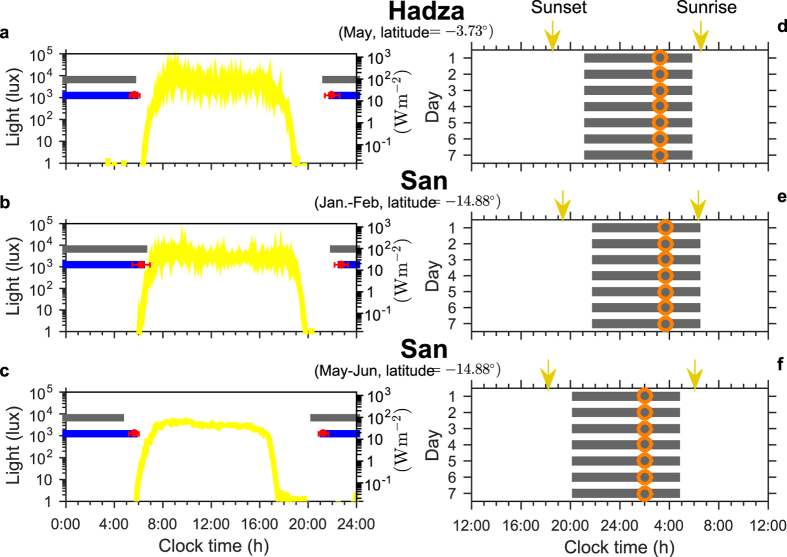
Timing of sleep and circadian rhythmicity when exposed to natural light/dark cycles. (**a–c**) Average light profiles (yellow) and sleep and wake timing (blue) (±standard deviation in red) for the Hadza and the San people who live a traditional hunter-gather lifestyle with no access to electrical light^22^. Sleep-timing as determined by the mathematical model using these same light profiles (grey) compare well with those determined in the field. (**d–f**) Computed sleep timing in relation to sunset and sunrise. Timing of the computed minimum of the circadian wake propensity is indicated by the orange circles. No fitting has been carried out for any of the model predictions: intrinsic circadian period is set to the human population average of 24.2 h; parameters for homeostatic rise during sleep and circadian amplitude have been set to be consistent with the age of the participants (see the Supplementary Material for further details on the relationship between parameters and age and the values of all other parameters).

Hunter-gatherers living near the equator, with no access to electric light, receive very bright day-time light (averaging approximately 4000 lux) and very low light levels before dawn and after dusk (less than 5 lux), when the only available light is from small fires. Objective assessment of sleep timing in three groups of hunter-gatherers has demonstrated that bed-times occur approximately two to four hours after sunset and wake-time follows on average 7.7 h later, approximately one hour before sunrise. Using the average measured light profiles^22^ as inputs to the model, with no constraints on the model’s sleep timing, the model closely replicates sleep duration and timing for the three groups (see Fig. 2). Thus, the model predicts that in the absence of access to artificial light our circadian system is entrained such that we wake at dawn. The simulations suggest that this is two to three hours after the minimum of circadian wake propensity (the maximum of circadian sleep propensity), as shown by the position of the orange circles in Fig. 1d,e.

### Access to self-selected evening light delays spontaneous sleep-wake timing in a dose-dependent manner that is greater when exposure to day light is reduced

**Figure 3:**
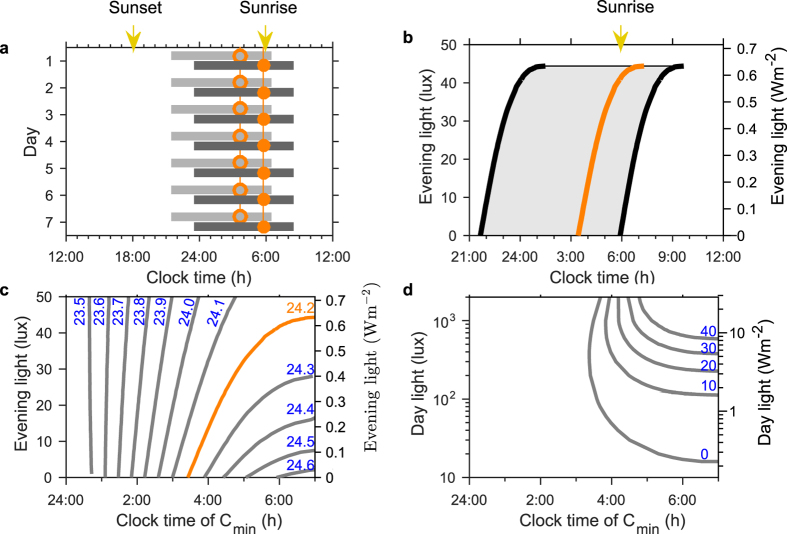
Sleep timing and timing of minimum circadian wake propensity during free days: dependence on levels of day and evening light. (**a**) Sleep timing for evening light of 5 lux (light grey) and 40 lux (dark grey) (day light 700 lux) with a photoperiod of 12 h centered on noon. The values of 700 lux and 40 lux were motivated by typical light levels reported for an industrialized society in the summer months^20^. The clock time of the minimum of the circadian wake propensity rhythm is shown by the orange circles. (**b**) Sleep timing as a function of different levels of evening light (day light 700 lux). At high levels of evening light, the model no longer synchronizes to a 24 h pattern. The orange curve marks the time of the minimum of the circadian wake propensity rhythm. (**c**) Timing of the minimum of circadian wake propensity as a function of evening light for different intrinsic periods. The average intrinsic period of 24.2 h has been picked out in orange. (**d**) Timing of the minimum of circadian wake propensity as a function of day light for different levels of evening light. While increasing evening light shifts timing later, increasing day light shifts timing earlier. Intrinsic period 24.2 h. Parameters for the homeostatic rise during wake and circadian set appropriate for age 30y in all panels.

The model predicts that increasing the brightness of artificial evening light delays the spontaneous timing of sleep (Fig. 3a,b). Here, sunset and sunrise have been set at 6:00 h and 18:00 h, respectively, and maximum day light levels have been set at values typically observed in industrialized societies^20^. Even at less than 40 lux of evening light, for those with an intrinsic period of 24.2 h, spontaneous wake-time is predicted to occur up to three hours after sunrise (Fig. 3b). Although sleep timing is delayed, the phase-relationship between wake-time and the minimum of circadian wake propensity is not markedly affected. Spontaneous wake-times consistently occur two to three hours after the circadian wake propensity minimum, as shown by the position of the orange circles.

The impact of evening light on spontaneous sleep timing is predicted to strongly depend on intrinsic circadian period (Fig. 3c). In approximately 80% of humans the intrinsic period is longer than 24 h and our simulated range of 23.5 h to 24.7 h covers approximately 99% of the intrinsic periods observed in healthy adults^3^. Hence our results imply that the impact of evening light will vary greatly between individuals, with those with longer intrinsic periods predicted to be very susceptible to the effects of evening light.

The effect of evening light on spontaneous sleep timing can, to some extent, be offset by increasing day-time light exposure (Fig. 3d). However, the relationship is highly nonlinear, with large increases in day-time light intensity needed to offset small increases in evening light intensity. This can be seen from the vertical distance between the lines for different evening lux values. For example, for a circadian wake propensity minimum time of 5:00 h, a factor of two increase in day-time light is required to compensate for each increase in evening light of ten lux. We note that both in Fig. 3b–d and in subsequent figures, we have computed data points at sufficiently fine intervals that using only linear interpolation the curves appear smooth, even in regions of high curvature.

### Socially imposed wake-times lead to accumulation of sleep debt during work days and later sleep timing during free days

**Figure 4:**
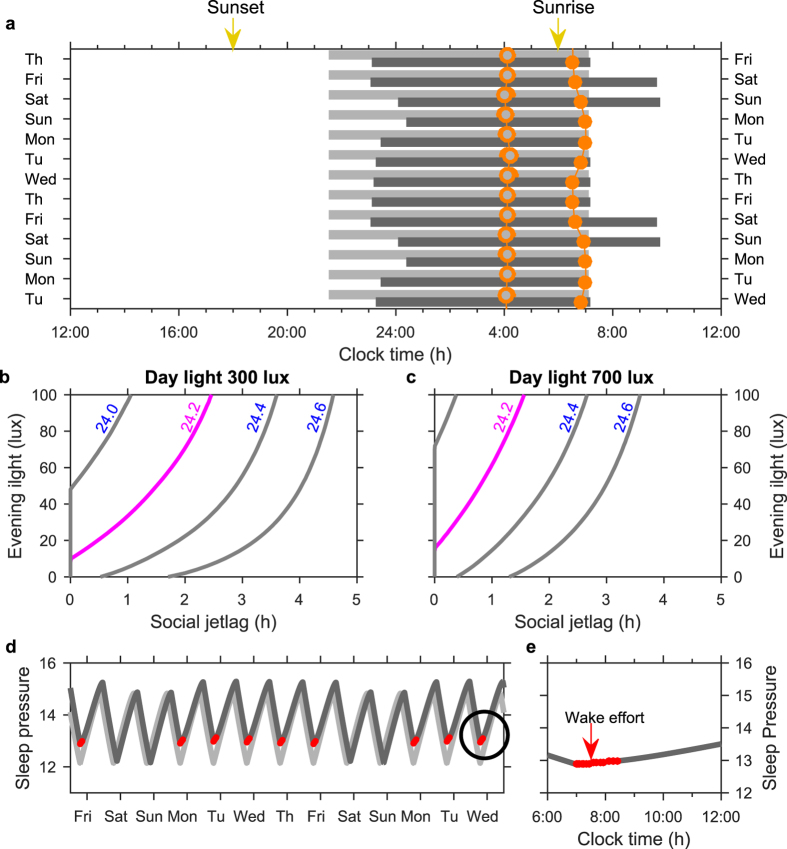
Effects of social constraints on sleep timing, social jet-lag and wake effort. (**a**) Sleep timing over a two-week period with a social constraint (forced wake time at 7:00 h) for five days of the week. The light/dark grey bars show sleep timing in the absence/presence of evening light. In this example, in the absence of evening light the model wakes spontaneously before the forced wake time. No social constraint is applied to bedtime, so sleep onset is spontaneous in both cases. The time of the minimum of the circadian wake propensity rhythm is indicated by orange circles. (**b,c**) Social jet-lag, as measured by the difference between wake time on Saturday and wake time during the week, for different values of evening light and different intrinsic periods. b day-time light 300 lux; c day-time light 700 lux. The values of 300/700 lux were motivated by typical values reported in the winter/summer months for an industrialized society^20^. (**d**) Daily time course of homeostatic sleep pressure across the two-week period in the absence/presence of evening light (light/dark grey). Red regions indicate times of wake effort. e Detail of sleep pressure on Wednesday morning, showing the region of wake effort. Parameters: day-time light level 300 lux typical for observed values during the winter months^20^. Evening light either 0 or 60 lux. Intrinsic period 24.3 h in (**a,d and e**), which is chosen to indicate a simulation with clear wake effort. Parameters for the homeostatic rise during wake and circadian amplitude set for age 17y.

Work schedules may override spontaneous wake timing. The model predicts that the effect of work schedules on the alignment of sleep-wake cycles with circadian rhythmicity strongly depends on artificial evening light. This is illustrated in Fig. 4a, where the social constraint of rising at (or before) 7:00 h on week days is imposed for two different light conditions: one with evening light and one without. For the example shown, in the absence of evening light, the model naturally wakes a few minutes before the 7:00 h alarm time, and sleep-wake timing is uniform across the week (light grey bars). Once access to evening light is introduced, the circadian wake propensity rhythm shifts later; bed-times are later and spontaneous wake-time occurs after 7:00 h. Because of the forced awakening during work days, differences between work days and weekends emerge, replicating the basic features of social jetlag, namely sleeping longer at the weekend to make up for accrued sleep debt during the week (dark grey bars) and sleep occurring in and out of phase with the circadian rhythmicity. The phase changes can be seen by the position of the orange circles that mark the minimum of the circadian wake propensity. When sleep is unconstrained, the minimum of the circadian wake propensity rhythm occurs two to three hours before wake (Fig. 4a). With the inclusion of social constraints, the position of the circadian minimum still occurs two to three hours before wake time at the weekend, but during the week, occurs much closer to wake time. With the social constraint, sleep on Sunday night is particularly short, because the circadian clock has been delayed as a result of sleeping in over the weekend, yet there is the requirement to wake at 7:00 h on Monday morning. Figure 4a simulates one particular set of parameters, but social jet-lag, quantified here as the difference between wake-time on Saturday and wake-time on week days, is dependent on both light exposure and physiological factors. Those with less light exposure during the day, more light exposure in the evening, longer intrinsic circadian periods, or other age-related physiological factors that result in later unconstrained wake-times, are predicted to experience more social jet-lag (see Fig. 4b,c). Note that insufficient light during the day can result in those with longer intrinsic periods having spontaneous wake times after the alarm time of 7:00 h and can result in social jetlag even in the absence of evening light. This can be seen both in Fig. 4b and c, and is consistent with the dependence of spontaneous sleep timing on day-time light, as shown for an intrinsic period of 24.2 h in Fig. 3d.

For the example shown in Fig. 4a, the alarm time occurs close to the minimum of the circadian wake propensity and the model predicts it will initially require effort to stay awake. This notion of ‘wake effort’^30^ corresponds to times when the model would naturally fall asleep if not kept awake by other wake-promoting factors. In the model, this is represented by an increased ‘drive’ to wake-promoting neurons, which could include the effect of caffeine or conscious effort devoted to remaining awake. The wake effort, which is different from the transient process of ‘waking up’ referred to as sleep inertia, is not required all day. As the day progresses, the wake promoting effect of the circadian wake propensity rhythm increases. Once it is sufficiently large to counterbalance the sleep-inducing effects of the homeostatic sleep pressure, wake effort is not needed. For the example shown, the wake effort lasts for approximately 1.2 h (regions in red in Fig. 4d, magnified in Fig. 4e). As for social jet-lag, the length of the wake effort period is dependent on physiological factors and day/evening light exposure: this is discussed further in the next section. Thus, during a week the model suggests many of us experience considerable wake effort during work days and this is associated with a cycle of accumulation and dissipation of slept debt across the week. Note that only at the weekend are levels of homeostatic sleep pressure comparable with the unconstrained case, see Fig. 4d, where the light grey line indicates the unconstrained case and the dark grey line shows the constrained case.

### In the presence of artificial light, delaying social cues such as work or school start-times leads to an initial reduction in social jet-lag which, in some cases, is only transitory

**Figure 5:**
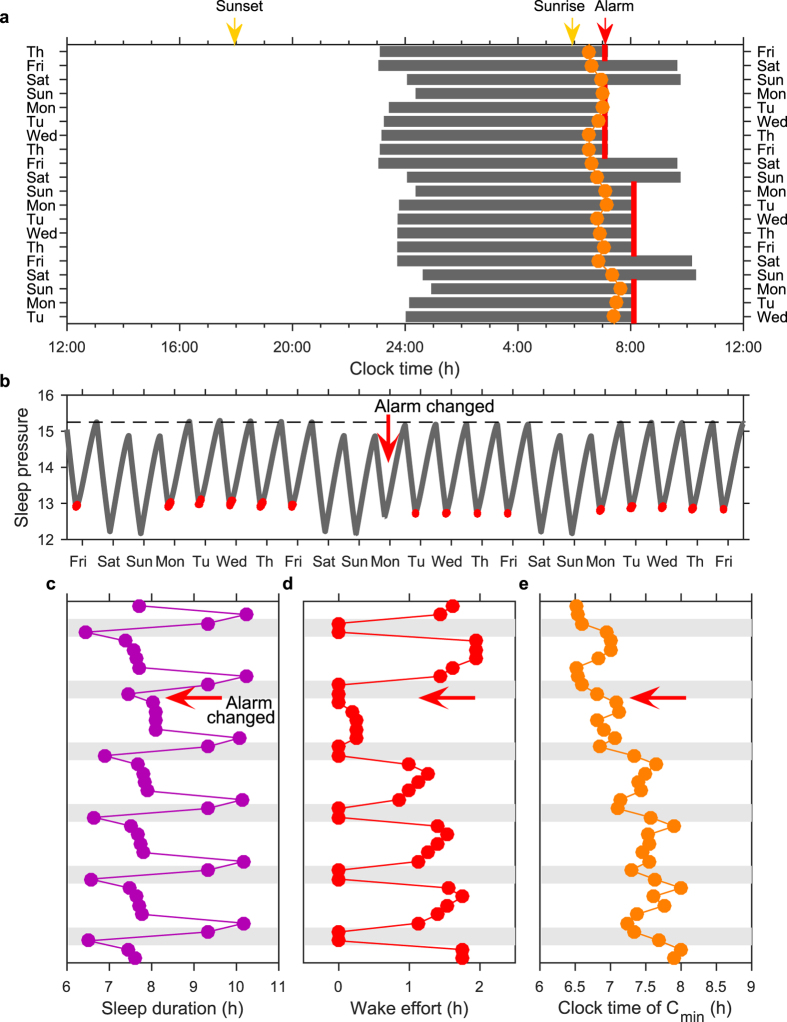
Short term effect of a change in alarm time. (**a**) Sleep timing over a three-week period where the alarm is set to 7:00 h for the first week and is changed to 8:00 h at the beginning of the second week. (**b,c,d and e**) show homeostatic sleep pressure, sleep duration, the minimum of the circadian wake propensity rhythm, and the number of hours per day of wake effort, respectively, over the same three-week period. Parameters are the same as for Fig. 4 a and d.

One suggested remedy for social jet-lag is to delay work/school start-times. However, changes to social constraints will also modify patterns of light exposure^31^. For example, delaying school start-time could delay both sleep-times and wake-times, leading to delayed circadian rhythms. This is shown in Fig. 5, where we model the impact of changing the week day alarm time from 7:00 h to 8:00 h, with sunrise occurring at 6:00 h. Sleep duration is significantly increased on the first day the alarm is changed and wake effort is no longer required (Fig. 5b,c). However, in the example shown, the increase in sleep duration and reduction in wake effort is largely transitory: after five weeks, the maximum increase in sleep duration on any one day is four minutes and mean week day wake effort is eight minutes. This is because the self-selection of evening light leads to a delay in the circadian wake-propensity rhythm (Fig. 5d).

**Figure 6:**
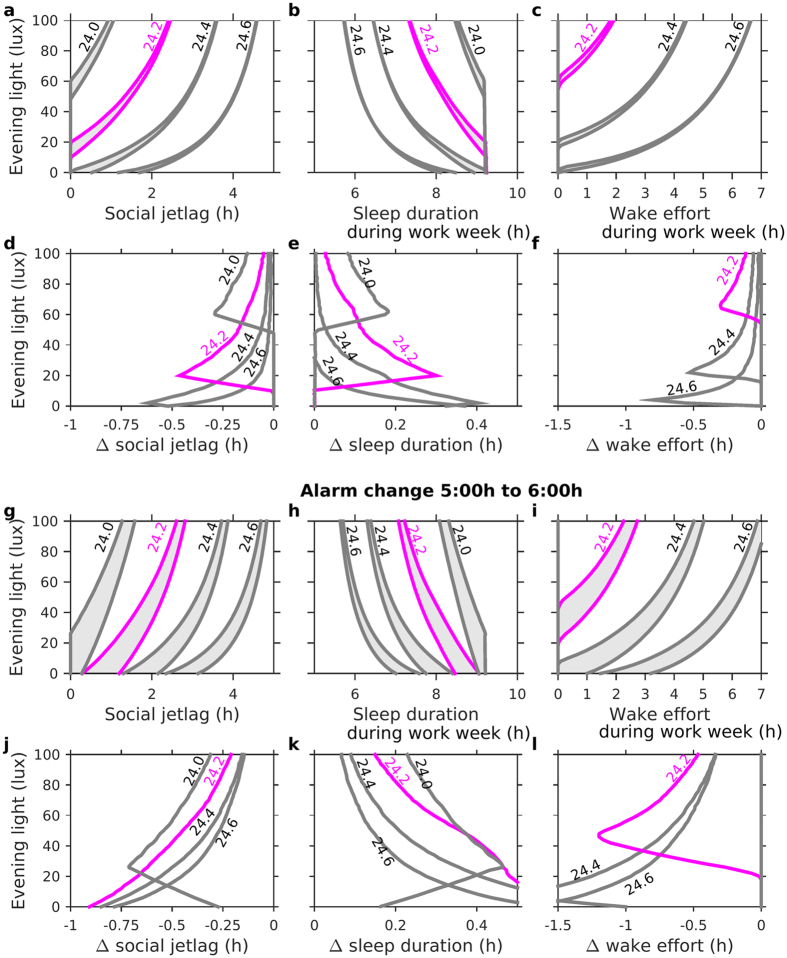
Long term effects of a change in alarm time. (**a–c**) Social jet-lag, mean week day sleep duration, and mean week day wake effort, respectively, before and 10 weeks after an alarm change from 7:00 h to 8:00 h for different intrinsic periods. The shaded regions highlight the magnitude of the change with the right-hand/left-hand edge of each shaded region representing the values before/after the change respectively. (**d–f**) The change in social jet-lag, sleep duration and wake effort, respectively, for different intrinsic circadian periods. (**g–l**) Show the analogous figures for an alarm change from 5:00 h to 6:00 h.

The effects of changing the social schedule depend on intrinsic circadian period and evening light. This is shown in Fig. 6a–c, where social jet-lag, sleep duration, and wake effort are all compared before and ten weeks after the alarm change, as a function of evening light and intrinsic period. The width of the grey band at a given light level indicates the change in social jet-lag/sleep duration/wake effort. The height of the grey band gives the reduction in evening light that would be needed to produce an equivalent effect. For example, with an intrinsic period of 24.2 h and 60 lux of evening light, the one hour delay in alarm time reduces social jet-lag from 1.76 h to 1.62 h, a reduction of eight minutes. An equivalent reduction could be achieved by decreasing evening light by 6 lux to 54 lux. Typically, the one hour delay in alarm time results in decreases in social jet-lag, increases in sleep duration, and a reduction in wake effort of substantially less than 1 h (Fig. 6d–f). Our results suggest that those with the longest intrinsic period have the most extreme social jet-lag, shortest sleep duration, and longest period of wake effort, yet they are also the ones who will be helped the least by a change in alarm time. For this group, reduction of evening light consumption is relatively much more effective.

The choice of changing the alarm time from 7:00 h to 8:00 h was motivated by proposed changes in the UK: typical school start-times for adolescents are currently 8:30 h to 9:00 h and a shift to 9:30 to 10:00 h has been mooted^32^. This follows an extensive and on-going debate in the USA. A recent review^33^, highlights the fact that, although as many as 80 school districts may have already shifted school timing for adolescents and some positive benefits have been reported, there is a need for further systematic research to quantify the effectiveness of this intervention. A key difference between the USA and the UK is that school start-times are substantially earlier in the USA, with some schools starting as early as 7:00 h. With the light profile used here, a shift in alarm time from 5:00 h to 6:00 h delays alarm time from an hour before sunrise to sunrise. Qualitatively, the results are similar to those for the change from 7:00 h to 8:00 h, but the social jet-lag is larger, sleep durations are shorter, wake effort lasts longer, and the change in alarm has a more beneficial effect. It is therefore possible that, for these very early start times, enough people would benefit to make a delay in start times worthwhile.

### Adolescents are particularly sensitive to the effects of evening light

**Figure 7:**
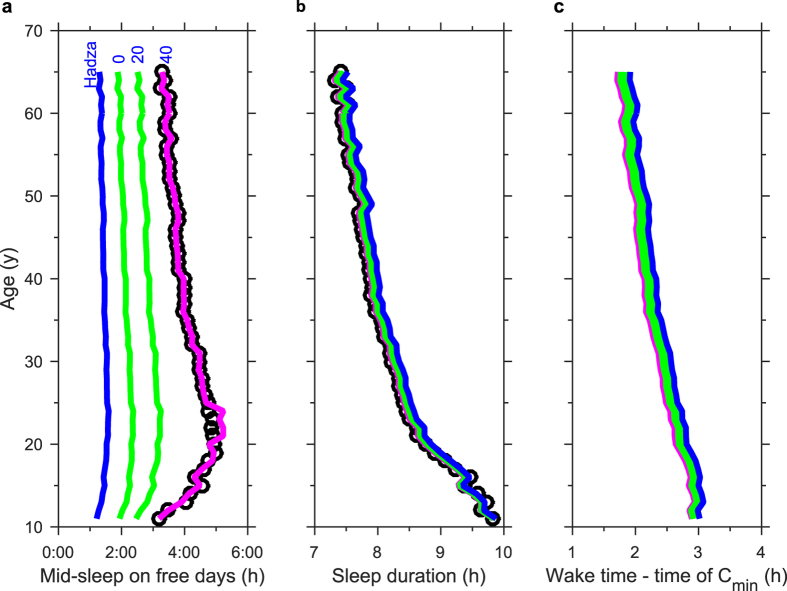
Sleep timing across the lifespan. (**a**) Sleep timing as measured by mid-sleep on days with no social constraints, (**b**) sleep duration, and (**c**) difference between wake-time and the minimum of the circadian wake propensity across the lifespan. Data for sleep timing and duration^34^,^70^ are shown as black circles. Results are shown for: day/evening light levels of 700/40 lux (purple); day/evening light levels of 700/20 and 700/10 lux (green); the light profile in Fig. 2a for the Hadza (blue)^22^. Further details on the age dependence of the circadian amplitude and the rise of the sleep homeostat during wake are given in Supplementary Material. The intrinsic circadian period is set to 24.2 h.

It is well-documented that preferred sleep timing changes across the lifespan, being latest at the end of adolescence^34^. Physiological changes across the lifespan that contribute to changes in preferred sleep timing may include a reduction in the rate of homeostatic rise during wake^35^,^36^ and a reduction in the circadian amplitude^37^,^38^. It has previously been shown that monotonically reducing the rate of homeostatic rise during wake and circadian amplitude reproduces age-related changes in sleep timing^29^. In Fig. 7 we show that monotonically reducing the rate of homeostatic rise during wake and circadian amplitude using our modified model using a realistic light profile also replicates age-related changes in sleep timing. The reported biological data^34^ considers times of the midpoint of sleep on ‘free’ days where there are no constraints on wake times. The comparison shown is therefore with simulations with no social constraints. Interestingly, however, this trend only matches the data that were collected from the general population when self-selected evening light is included. Keeping the same maximum day light level of 700 lux, but removing evening light, the predicted differences in sleep timing with age are modest. Using mean light data from the Hadza^22^, shown in Fig. 2a, yields a similar prediction, although the higher intensity of day light systematically shifts the sleep of all age groups to earlier times. The simulations also show that the interval between wake time and the minimum of the circadian wake propensity becomes smaller with increasing light exposure and with age. The latter observation is in accordance with empirical observations^18^. Overall the simulations imply that the profound delay in sleep observed in adolescents in modern industrialized societies is largely a result of our ability to self-select patterns of light exposure.

### Theoretical underpinning and model robustness

**Figure 8:**
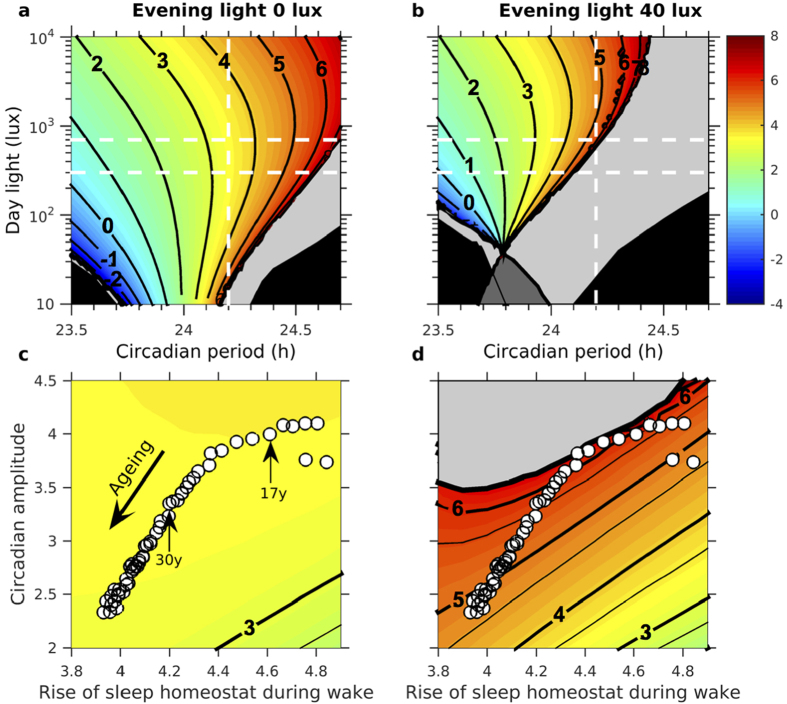
Resonance tongues with and without self-selected evening light. (**a** and **b**) The colored balloon-shaped regions show the regions of entrainment by light, with contours indicating different phases of the minimum of the circadian wake propensity rhythm for evening light 0/40 lux respectively. In the light grey region, light alone is insufficient to entrain the model but the model can be entrained to an average of 24 h by social constraints applied for five days per week. In the black regions the model does not entrain even with social constraints. In the dark grey region, the model entrains to sleeping during the day. The thin black line splits this dark grey in two, to the right of this line, in the presence of social constraints the model is entrained to a schedule of sleeping at night. Age parameters set to 30y. The white dashed lines are to help guide the eye and mark the average intrinsic period of 24.2 (vertical line) and the levels of 700 and 300 lux (horizontal lines) that are used in many of the other simulations in the paper. (**c,d**) Circadian phase as a function of the parameters modelling the rate of homeostatic rise during wake and circadian amplitude. Circles indicate the position of the age-dependent parameters chosen to match reported data^34^,^70^ with 700 lux during the day. c Evening lux 0. d Evening lux 40.

One might argue that the effects of artificial light presented here are model-dependent, and that varying the model will vary the results. However, any model that describes circadian rhythmicity as an oscillator entrained by light will show similar phenomena. This is because an entrainable oscillator necessarily has a resonant ‘tongue’. The tongue describes how the combination of parameters (intensity of light and intrinsic period) affects entrainment. The rainbow-colored balloon shaped areas in Fig. 8a represent combinations of parameters for which the circadian oscillator is phase-locked (entrained) to the 24-h light-dark cycle. In the center of the tongue, small changes in the light profile have little impact on the circadian phase of entrainment. Whereas near the edges of the tongue the circadian phase of entrainment is highly sensitive to changes in the light profile. The sensitivity to changes in light at the tongue margins is a consequence of approaching a bifurcation (specifically, the transition from entrained to non-entrained states occurs via a saddle-node bifurcation and local to the saddle-node point any phase marker has a square-root behavior). The presence of the saddle-node bifurcation means that, near the tongue margins, entrainment takes a long time (so-called critical slowing-down).

Self-selection of light results in a reduced entrainment region, as shown in Fig. 8b for 40 lux of self-selected evening light in addition to the day-time light used in Fig. 8a. The minimum of the tongue is now at 40 lux: if the day light and the evening light are identical then there is no daily rhythm of light to entrain the model. It is this tongue structure that underpins the results shown in Figs 3, 4, 5, 6. The position of the white dashed lines in Fig. 8a,b are to guide the eye and mark the position of the average intrinsic period of 24.2 h and the levels of 300 and 700 lux used in many of the simulations. The position of these lines in Fig. 8b highlights the importance of social constraints to maintain entrainment in the absence of sufficient differentiation between day light and evening light. In the light grey region, light alone is insufficient to entrain the model to 24 h, but the model can be entrained to a weekly schedule by social constraints. In other words, the simulations suggest that in the presence of self-selection of artificial light, synchronization to the 24 h day is compromised in a large section of the population, particularly when daylight light exposure is low. Specifically, using the measured distribution of intrinsic periods in humans^3^, for 300 lux of day light and 40 lux of evening light, our simulations suggest that more than 60% of the population require social constraints to remain entrained. When social constraints are needed for entrainment, delaying alarm times does not greatly reduce social jet-lag.

The position of the tongue-shaped region is dependent on other model parameters. As discussed above, variation in sleep timing with age can be explained by decreasing homeostatic rise during wake and decreasing circadian amplitude. Variations in these two parameters result in a shift, first towards the edge of the tongue and then away from it. This is illustrated in Fig. 8d for the light profile that was used to match the sleep timing data in^34^, and shows the delay of approximately two hours in sleep timing from ages 11y to 20y and subsequent shift back to earlier sleep at later ages. The fact that this variation in sleep timing is largely eliminated if evening light is removed, as shown in Fig. 7, is a consequence of the increase in size of the entrainment region for decreasing evening light: Fig. 8c shows the change in phase that results from the same variation of homeostatic rise during wake and circadian amplitude with no evening light.

## Discussion

Mechanisms of entrainment of circadian systems to the 24 h day have evolved under conditions under which the daily light-dark cycle was imposed by the Earth’s rotation and organisms had no behavioral means to extend the photoperiod. This fundamentally changed with the invention of methods to artificially produce bright light and has had a profound impact on the timing of sleep by giving us greater flexibility in social schedules. The consequent changes in patterns of light exposure received by the circadian clock from this behavioral feedback loop has previously been referred to as a ‘zeitnehmer’^39^. Our mathematical analysis provides the first framework for quantitatively predicting how this phenomenon will play out in the real world in humans in interaction with age-related or individual differences in physiology. This framework is underpinned by the resonant ‘tongue’ structures shown in Fig. 8a,b. Tongue-like structures generically occur in forced oscillator systems^40^,^41^ and have previously been described in models of circadian pacemakers^42^,^43^,^44^, but the inclusion of a second oscillatory process, the sleep homeostat, and the behavioral feedback both introduce novel additional dynamical features which have not previously been explored.

This theoretical structure provides a unifying framework within which to understand much recent work on sleep timing. For example, consistent with our simulations, recent field studies show that circadian phase, as indexed by the onset of nocturnal melatonin secretion, is much earlier and group variation in melatonin onset is greatly reduced when modern humans go camping and forego access to artificial light^21^. Access to artificial light is correlated with later sleep onsets^45^,^46^,^47^ and artificial outdoor lighting levels correlate with sleep complaints and delays in sleep timing^48^. Evening light (including light from electronic devices) suppresses and delays melatonin, reduces evening sleepiness, delays sleep timing, and affects sleep structure as well as the spectral composition of the EEG during sleep^11^,^49^,^50^.

Consequently, and also consistent with our simulations, spontaneous sleep timing in industrialized societies is after sunrise^51^, and many people have difficulty waking for work/school^52^. The mismatch between school/work start times and preferred sleep timing (social jet-lag), is worse in individuals who report lower levels of day light exposure^31^, and in individuals with longer intrinsic periods^53^. Social jet-lag is worse in individuals living on the western border of a time-zone compared to those to the East (See Fig. S2) due to differences in solar timing^54^ and delaying social constraints leads to some positive but small and possibly transitory effects on sleep duration, social jet-lag and wake effort^33^,^55^.

Yet the same theoretical structure that agrees with these recent observations, makes some surprising predictions. Namely: that the effectiveness of ‘countermeasures’ such as delaying social schedules without concerted changes in an individual’s light consumption behavior is likely to be limited; that endeavoring to improve sleep timing by shifting alarm time will necessarily lead to a further delay of the circadian sleep propensity rhythm; that delaying social schedules is relatively more effective in individuals who are already early types (shorter intrinsic periods) or on schedules that are very early relative to solar time (e.g., US school schedules) so that those who are affected the most by social jetlag will be benefitted the least; that a relatively small reduction in evening light is a similarly effective intervention to changing the alarm from 7:00 h to 8:00 h; that the reduction in the rate of homeostatic rise during wake across adolescence^35^ renders adolescents particularly sensitive to the effect of evening light, and that in hunter-gatherer societies without access to evening light, large differences in sleep timing across the lifespan will not be observed.

If, in hindsight, these predictions seem obvious, it should be remembered that much of the literature on preferred sleep timing, so-called chronotype, makes no mention of light. Consequently, chronotype is often reported as if it were trait-like rather than a combination of intrinsic and extrinsic factors^31^. This then leads to the misconception that adolescents are ‘programmed’ to wake up late. In the debate on changing school start times, there is little mention of light consumption behavior or the light environment. Clock time is used as a measure of whether a start time is ‘early’, yet some time zones span many degrees of longitude so what is ‘early’ on the Western edge of a time zone will not be so early on the East. Adolescents may also experience very different day-time light levels depending on their geographical location. Consequently, changes to school start times seem ill-advised without full consideration of the light consumption behaviour. It may be that the different conclusions reached^55^ on the relative effectiveness of moving school start times can be explained by differences in the light environment.

We note that we have not explored the effects of seasonal changes in photoperiod or societal choices that influence decisions to go to bed such as the decision to stay up late on free days in the knowledge that there is no constraint on wake time. In addition, we have assumed the same light sensitivity and the same rules for self-selection of artificial light for all individuals and ages although recent studies suggest the circadian system may be more sensitive to light during adolescence^56^ and adolescents are the group most prone to behavior that results in greater evening light from devices^57^. These factors may therefore further exacerbate delayed sleep and circadian timing in adolescence. We have also not considered variations in the spectral composition of artificial light, but a switch to LED based lighting which has a greater blue component will lead to a greater circadian effect at comparable illuminance levels, so we may therefore have under-estimated the effects of artificial light.

We also note that by reducing light during the day and increasing light during the evening, we are reducing the strength of the light-dark cycle as a zeitgeber. This increases the relative importance of other zeitgebers, both social and metabolic.

Our simulations (Fig. 8b) suggest that for many individuals who spend much of their time indoors, entrainment to 24 h is compromised in the absence of social constraints. These observations may be relevant to our understanding of circadian rhythm sleep disorders such as non-24h (i.e. free running type) in sighted individuals. Caffeine consumed in the evening shifts circadian phase to later times^58^, and this effect is likely to interact with the effects of light exposure.

Overall, our results highlight the need for further research to understand the effect of light, including its spectral composition, on the circadian timing system and on sleep timing. Since a characteristic of modern sleep is sleeping in-and out-of-phase with our circadian rhythm, there is a need to develop biomarkers for circadian phase that do not use sleep-timing as a proxy. Any systematic study of sleep timing should necessarily consider light exposure as a factor, ideally including the measurement of spectral composition. Furthermore, not only clock time but also position in the time zone, season, and solar time should all be recorded^54^. Since the decision to go to bed is under social control and not solely determined by sleepiness, there is also a need for further investigation of the reasons for selection of a particular bed-time.

Our work confirms and implies that: we should educate teachers, parents, and students on sleep hygiene and the role of light; that we should advocate brighter light in classrooms and offices, or access to natural light in the morning; that we should minimize light in the evening as far as possible; and that light interventions may be more effective at reducing social jet-lag than changing school/work start-times.

From a global development and management of urban environments perspective, the light environment and light exposure should be considered as a major determinant of human circadian biology and health. For example, policy makers could encourage building design that incorporates access to natural light and consider the impact on human sleep and the environmental impact on other species^59^ of the increasingly pervasive nature of artificial light in the evening^60^.

## Methods

We use a modified version of the mathematical model originally described in^28^ that incorporates three core elements of sleep-wake regulation: (i) mutual inhibition between wake-promoting and sleep-promoting neurons, resulting in sleep and wake states^61^; (ii) switching between sleep and wake states as a result of the interaction of homeostatic sleep pressure and an endogenous circadian rhythm^14^,^62^; (iii) an external light signal that forces the natural circadian oscillator^12^ and is only perceived during wake to mimic the gating effect of the eyelid. Variants of this model have been used to investigate: chronotype^28^, internal desycnchrony^63^ non-rotating^64^ and rotating shift schedules^65^, and age-related changes to sleep timing and duration^29^. This model has a close relationship with the original two-process model^66^, and similar results would result from considering a two-process model with the phase and amplitude of the circadian rhythm determined by an external signal gated by the eye as in the model used here. The full equations and details of the parameters are given in the Supplementary Material.

Two kinds of light profiles are used: first, average light profiles measured in three different hunter-gatherer groups living in a natural environment with no access to artificial light^22^. These light profiles have been provided to us courtesy of Prof. Siegel. Second, we consider a light profile that is similar in shape/overall level to the average light profiles measured in modern humans living in the Surrey area (UK, latitude 51.1-51.5^◦^N, longitude −0.8–0.1^◦^W) using similar methodology to that used in the hunter-gatherer groups but with a photoperiod of approximately 12 hours centered on noon.^20^. The particular day-time light levels of 700/300 lux are representative of light levels that were measured in the summer/winter respectively. This light profile reaches a maximum during day-light hours and flattens off to a lower level post-sunset/pre-sunrise. The time at which light is turned on/off is determined by when the model wakes up/goes to sleep and is thus ‘self-selected’. Many of the results focus on how sleep duration and timing vary as the day-time maximum level and the post-sunset/pre-sunrise level are varied. Since the dominant effect of the self-selected light is post-sunset rather than pre-sunrise, it is referred to as evening light. Light data from the hunter-gatherer study^22^ and that underlying the circadian model^12^ were measured in lux. For comparison we have included a scale in W/m^2^, where a factor of 70 between lux and W/m^2^ has been used: this a value that is at the lower end of the range of values for natural light and the upper end of the range of values for light from artificial sources^67^. We note that the model was developed using light levels measured at eye level, which in general will be higher than the light levels that were measured at the level of the wrist^20^. Also, we note that it is increasingly clear that lux, which reflects the efficacy by which the rod and cone system influence our visual perception of the external world, is not an accurate unit for describing the impact of light on the circadian system. In addition to the classical cone and rod photoreceptive system, a ganglion-based melanopic system contributes to the effects of light on the circadian system^4^. Light with a wavelength close to 480 nm (blue light) is particularly alerting and delays the onset of melatonin more than light of the same intensity at other wavelengths^11^,^68^,^69^. The light forcing part of the mathematical model is based on data collected prior to the discovery of the ganglion-based melanopic system, consequently it is likely that our results underestimate the role of evening light: as yet, there is insufficient data to update the model in a quantitative manner.

The model parameters are listed in Table 1 in the Supplementary Material. It has been shown that sleep-timing and duration from ages 11 to 65^34^,^70^ can be replicated by varying the circadian amplitude and the rate of rise of the sleep homeostat during wake that models the need for sleep^29^. To investigate age effects, we therefore vary the two parameters that model circadian amplitude and the rate of rise of the sleep homeostat during wake. We also vary the endogenous circadian period, since there is a literature on the changes of sleep timing with endogenous circadian period. Values for all other parameters for sleep/wake regulation and the circadian systems are the same as those used previously^28^.

Two different schedules are considered: either unconstrained, where sleep and wake are determined purely by the model; or socially constrained, where a weekly schedule is imposed. During this weekly schedule, the model is required to be awake at a particular alarm time (7:00 h unless otherwise specified) on Monday to Friday and wake timing is selected by the model on Saturday and Sunday. Further details on how this is implemented are given in the Supplementary Material Note that since photoperiod is set to approximately 12 hours centred on noon, sunrise and sunset are approximately 6:00 h and 18:00 h respectively.

We note that the coupled oscillator nature of the model leads to the tongue-like structures that provide the underpinning framework for all the results. There are three major differences between our mathematical model and earlier work on circadian oscillators. (i) Our model contains a circadian oscillator that is entrained by light that, in turn, entrains a sleep oscillator. Consequently, entrained circadian rhythms do not necessarily result in entrained sleep patterns^63^. The presence of both a circadian and a sleep oscillator also implies that the phase relationship between the two can be non-trivial, making sleep timing an imperfect proxy for circadian phase and vice versa. (ii) The sleep oscillator determines the forcing signal received by the SCN: during sleep, when the eyes are shut, it is assumed that no light enters the eye. Hence, even if the light-dark signal is strictly periodic, the forcing received by the circadian oscillator is only periodic when sleep is entrained. This self-selection of light results in a reduced entrainment region, as shown Fig. 8b for 40 lux of self-selected evening light in addition to the day-time light used in Fig. 8a. The minimum of the tongue is now at 40 lux: if the day light and the evening light are identical then there is no daily rhythm of light to entrain the model. (iii) The inclusion of a sleep oscillator enables us to consider the impact of social constraints on sleep debt, sleep variability and the effort to wake up since these are associated with the phase relationship between the sleep-wake cycle and circadian rhythmicity.

### Data Availability

Details of the data and how to request access are available from the Surrey Research Insight Open Access Repository DOI: 10.15126/surreydata.00813727.

## Additional Information

**How to cite this article:** Skeldon, A. C. *et al*. The effects of self-selected light-dark cycles and social constraints on human sleep and circadian timing: a modeling approach. *Sci. Rep.* **7**, 45158; doi: 10.1038/srep45158 (2017).

**Publisher's note:** Springer Nature remains neutral with regard to jurisdictional claims in published maps and institutional affiliations.

## Supplementary Material

Supplementary Material
