# Supplementary material for: The effects of self-selected light-dark cycles and social constraints on human sleep and circadian timing: a modeling approach
Source: Sci Rep. 2017 Mar 27;7:45158. doi: 10.1038/srep45158 (PMC5366875; doi:10.1038/srep45158)
Supplement: Supplementary Material [file srep45158-s1.pdf]

The effects of self-selected light-dark cycles and social constraints on human sleep and circadian timing: a modeling approach

Supplementary material

Anne C Skeldon[1,*], Andrew J K Phillips[2], and Derk-Jan Dijk[3]

[1]University of Surrey, Department of Mathematics, Guildford, GU2 7XH, UK
[2]Harvard Medical School, Division of Sleep and Circadian Disorders, Brigham and Women's Hospital, USA
[3]University of Surrey, Surrey Sleep Research Centre, Guildford, GU2 7XP, UK
[*]a.skeldon@surrey.ac.uk

Equations

The results shown in Figs 1-8 come from numerically integrating a modified version of the Phillips-Chen-Robinson (PCR) model[1] using the stiff solver ODE15s in MATLAB[2]. Full equations, parameter choices and the method for implementing social constraints are discussed below.

Phillips-Chen-Robinson (PCR) model

The PCR model itself combines two earlier models, one describing switching between sleep and wake states as a result of a drive consisting of homeostatic and circadian components[3], and one describing the entrainment of the circadian system by light[4]. Specifically, sleep and wake states occur as a result of mutual inhibition between sleep promoting and wake promoting neurons as described by equations for their mean electric potential, V_v and V_m, respectively,

$$\tau_v \frac{dV_v}{dt} = -V_v - \nu_{vm} Q_m + D_v, \quad (1)$$

$$\tau_m \frac{dV_m}{dt} = -V_m - \nu_{mv} Q_v + D_m. \quad (2)$$

Here,

$$Q_{v,m} = \frac{Q_{\max}}{1+\exp[-(V_{v,m}-\theta)/\sigma]},$$

describes the sigmoidal relationship between the potential and the firing rate of the neurons, $Q_{v,m}$. The parameter $Q_{\max}$ is the maximum possible firing rate, θ is the value of the potential $V_{v,m}$ at $Q_{v,m} = Q_{\max}/2$ and σ determines the width of the sigmoid. The parameters $\tau_{v,m}$ give the typical timescales of the neuronal process and the parameters $\nu_{vm,mv}$ weight the input from population m to v and v to m respectively. The $D_{v,m}$ are 'drives'. The drive to the wake promoting neurons, D_m is fixed. The drive to the sleep promoting neurons, D_v, consists of homeostatic and circadian components,

$$D_v = A_v - \nu_{vc} C(t) + \nu_{vh} H(t),$$

where $H(t)$ describes the homeostatic sleep pressure modelled as the level of some somnogenic chemical such as adenosine, and $C(t)$ represents the circadian rhythm of the suprachiasmatic nucleus (SCN), the so-called master clock. The parameter ν_{vh} describes the ability of H to promote sleep. As discussed further below, $C(t)$ is approximately sinusoidal with amplitude close to one, so ν_{vc} gives the amplitude of the circadian wake propensity rhythm. The term A_v is a constant background inhibitory input to the sleep promoting neurons. The homeostatic process is given by

$$\chi \frac{dH}{dt} = -H + \mu Q_m. \quad (3)$$

Here, H/χ is the rate of removal of somnogenic chemical and $\mu Q_m/\chi$ is the rate of production. The circadian rhythm $C(t)$ is modeled by a forced van der Pol oscillator, where the forcing represents the light intensity dependent signal from photoreceptors in the eye. Specifically,

$$\kappa\frac{dx}{dt} = \gamma\left(x-\frac{4x^3}{3}\right)-y\left(\left(\frac{24}{f\tau_c}\right)^2+kB\right), \tag{4}$$

$$\kappa\frac{dy}{dt} = x+B, \tag{5}$$

$$\frac{dn}{dt} = \lambda\left(\alpha_0\left(\frac{\tilde{I}}{I_0}\right)^p(1-n)-\beta n\right), \tag{6}$$

where

$$B = \alpha_0(1-n)(1-bx)(1-by)\left(\frac{\tilde{I}}{I_0}\right)^p, \tag{7}$$

$$\tilde{I} = \mathscr{H}(Q_m-Q_{th})I(t). \tag{8}$$

Here, x and y are the variables for the van der Pol oscillator and n is the fraction of activated photoreceptors. The term B describes the forcing that occurs as a result of the action of light of intensity I on photoreceptors. This forcing term takes into account the fact that the circadian oscillator is known to be more sensitive to light at some phases than others and also that there is a saturation effect: the circadian system is more sensitive to changes in light intensity than to the absolute value *per se*. The sensitivity to phase is determined by the term $(1-bx)(1-by)$ in equation (7). The rate of saturation of photoreceptors is given by $\lambda\alpha_0\left(\frac{\tilde{I}}{I_0}\right)^p+\lambda\beta$ and the rate for decay is given by $\lambda\beta$, where the values of $\lambda, \alpha_0, I_0, p$ and β have been determined from experimental data[5] . The parameter τ_c is the intrinsic period, γ is the stiffness of the oscillator. Note that the intrinsic period of the van der Pol oscillator is dependent on light intensity since for diurnal species intrinsic period is shorter for higher light intensities (Aschoff's rule). This dependence of the intrinsic period on light intensity is controlled by the parameter k in equation (4). The circadian rhythm of the SCN, $C(t)$, is then given by

$$C(t) = \frac{1}{2}(1+y). \tag{9}$$

The model for the circadian rhythm of the SCN given in equations (4) to (7) has been shown to produce a good match to human phase response data[4].

In coupling the circadian rhythm to the sleep/wake model given in equations (1) to (3), the PCR model assumes that the light signal is gated by the eye. This is represented by the Heaviside function in equation (8), which means that light impinging on photoreceptors is zero if the firing rate of wake promoting neurons, Q_m, is greater than a threshold value Q_{th}. Originally,[1] light intensity was modelled as a sinusoidal input

$$I(t) = \frac{I_A}{2}(1+\cos\omega t), \tag{10}$$

with $I_A = 10000$ lux. With the typical parameters used, this gave sleep-wake cycles with sleep occurring between approximately 23:00h and 7:00h.

Modified PCR model

Typical measured light profiles are far from sinusoidal. Motivated by real world light profiles[6,7], here we have taken

$$I(t) = l_2+\frac{(l_1-l_2)}{2}\tanh(c(t-s_1))-\tanh(c(t-s_2)). \tag{11}$$

This switches between a level l_1 during core day light hours and l_2 at other times, where the switch from l_2 to l_1 occurs around $t = s_1$ and from l_1 to l_2 around $t = s_2$. The speed of the switch is determined by the parameter c. Parameter values for s_1 and s_2 were chosen to give daylight durations of approximately 12h centred on noon, equivalent to daylight duration at the equinoxes. The parameter c was chosen to give a steepness comparable to average measured profiles for summer (Apr-Aug) and winter Nov.-Feb.)[6]. Increasing the parameter c has two consequences for the light profile. Firstly, it makes the switches from light to dark and dark to light occur more rapidly and, secondly, it lengthens the photoperiod. The net effect is to shift sleep timing earlier. We have taken a pragmatic approach of fixing c and the photoperiod to realistic values. The light profiles for typical

values used in the paper ($l_1 = 700$ and 300, $l_2 = 40$) are shown in Fig. S1a and b. The two examples shown in Fig. 1 of the paper correspond to $l_1 = 4000, l_2 = 0$ (shown in black) and $l_1 = 300, l_2 = 60$ (shown in red).

Using realistic light intensity profiles for $I(t)$ in equation (8), it is difficult to reproduce typical observed values for both sleep duration and timing using equations (1)-(9). Since the van der Pol oscillator variables x and y cannot currently be directly related to the sleep/wake propensity rhythm, we have used a phase-shifted version of equation (9),

$$C(t) = \frac{1}{2}\left(1 + 0.80y - 0.47x\right). \tag{12}$$

The wake propensity rhythm is then given by $\nu_{vc}C(t)$. This results in sleep-wake cycles that with typical modern day light profiles can be matched to typical timings and sleep durations across the lifespan which the original model cannot. All the parameters values used are listed in Table S1. The sleep/wake regulation and circadian parameters are the same as those used in the original version of the model[1]. A typical solution for equations (1)-(8) using $C(t)$ as given by equation (12) and light profile given by equation (11) is given in Fig. S1.

**Sleep/wake regulation parameters**					
$Q_{\max}$	100s^{-1}	θ	10mV	σ'	3mV
ν_{vm}	2.1mVs	ν_{mv}	1.8mVs	ν_{vc}	$2-4.5$mV
ν_{vh}	1mVnM^{-1}	A_m	1.3mV	A_v	-10.2mV
τ_m	10s	τ_v	10s	χ	45h
μ	$3.8-4.9$nMs	Q_{th}	1s^{-1}		
**Circadian parameters**					
κ	$\frac{12}{\pi}$h	γ	0.23	f	0.99669
τ_c	23.5–24.7h	k	0.55	α_0	0.16
I_0	9500 lux	β	0.013	b	0.4
p	0.6				
**Light parameters**					
c	1/6000	l_1	0–10000 lux	l_2	0–100 lux
s_1	7.5h	s_2	16.5h		

Table S1. Parameter values for the Phillips-Chen-Robinson model.

Age dependence of parameters

In a post-industrial society, systematic changes in sleep timing and duration across the lifespan have been reported[8,9]. It has been suggested that in older people the homeostatic sleep pressure during wake increases at a slower rate than in younger people[10], and also that the amplitude of the circadian rhythm is reduced[11]. In the mathematical model presented in equations (1)-(10), these two changes can be modelled by reducing the parameters μ and ν_{vc}. It has been shown that reducing μ and ν_{vc} can indeed account for the changes in sleep-timing and sleep duration[12].

We have reproduced this result with our modified version of the PCR model and with a light profile that is more realistic. Specifically, using the light profile given in equation (11) above with $l_1 = 700$ lux, $l_2 = 40$ lux and s_1 =8:00h and s_2 =17:00h. The match to the data results in values for age 30 of $\mu = 4.20, \nu_{vc} = 3.37$ and for age 17 of $\mu = 4.60, \nu_{vc} = 4.00$, the two representative ages used in the simulations shown in Figs. 1-8.

For comparison with previous literature, we note that the two process model seeks to describe the time course of the power in the delta band (typically 1-4Hz) in EEG signals during sleep which is a biomarker for the homeostatic process. Typically, so-called process S is modelled by the differential equations

$$\chi_w \frac{dS}{dt} = -S + \tilde{\mu},$$

during wake and

$$\chi_s \frac{dS}{dt} = -H,$$

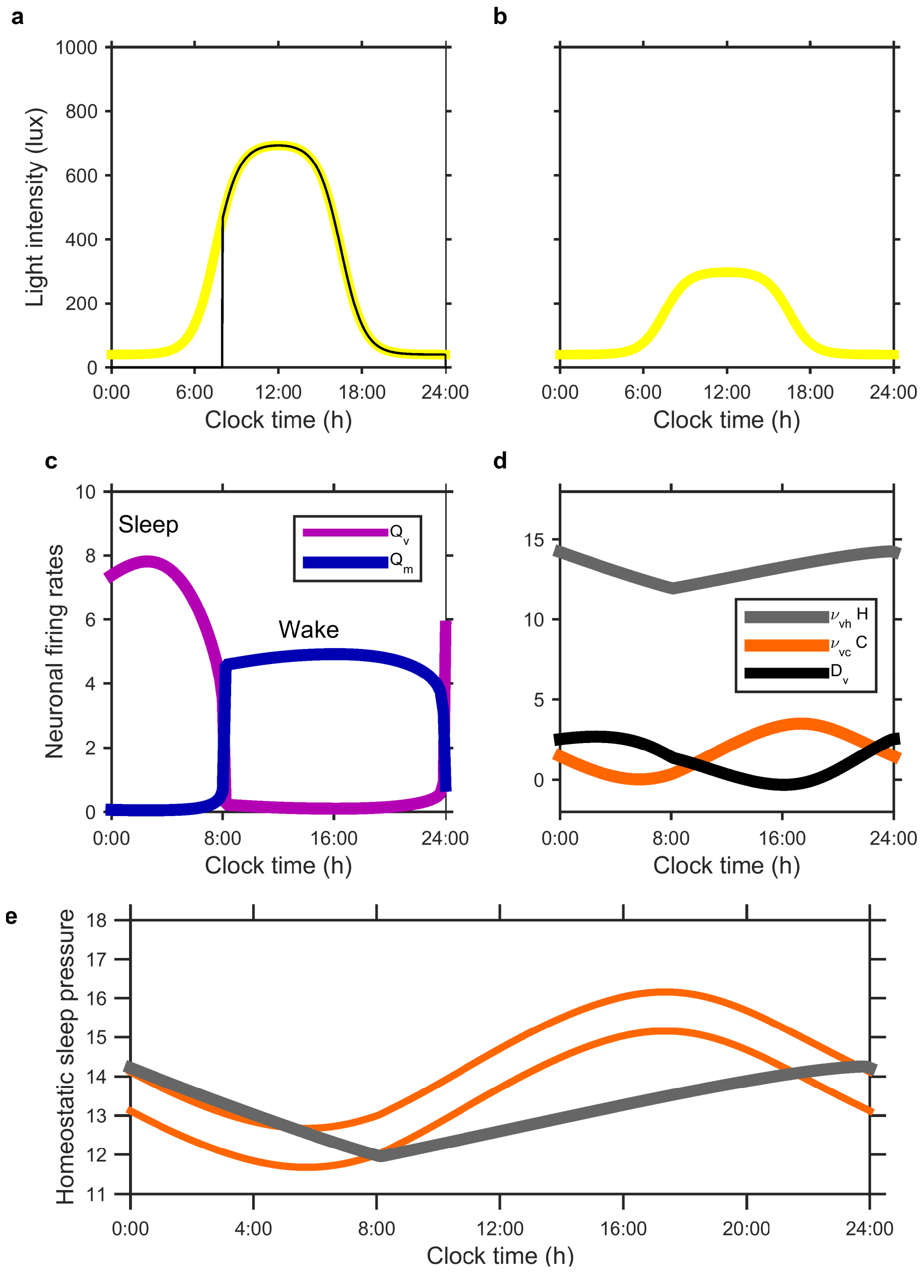

Figure S1. **a** and **b** typical light profiles (yellow) given by equation (11) for $l_1 = 700, 300$ respectively; $l_2 = 40$ in both cases. All other parameters are given in Table S1. In a, the thin black line shows the light profile gated by the sleep-wake cycle for the particular case shown in c-e. **c** The firing rates for the wake promoting and sleep promoting neurons, Q_m and Q_v respectively. **d** The homeostatic sleep drive, $\nu_{vh}H$ (dark grey),, the circadian wake propensity $\nu_{vc}C$ (orange) and the sleep drive D_v (black). **e** The data re-plotted in the form of the two process model, showing the homeostatic sleep presssure decreasing during sleep until it reaches the lower threshold, then increasing during wake until a spontaneous switch to sleep occurs when the homeostatic sleep pressure reaches the upper threshold. The upper and lower thresholds are modulated by the circadian wake propensity rhythm. For c-e, the light profile illustrated in a was used; $\mu = 4.20, \nu_{vc} = 3.37, \tau_c = 24.2$. All other parameter values are given in Table S1.

during sleep. There is an arbitrariness in the scale of S so that $\tilde{\mu}$ is normally taken to equal one. The parameters χ_w and χ_s are measured from the EEG. In general $\chi_w \neq \chi_s$. The experimentally measured reduction in rate of increase of homeostatic sleep pressure is normally reported as an increase in the value of χ_w.

In the PCR model, in both its original and our modified form, the homeostatic sleep pressure is modelled by equation (3). Since during wake Q_m is approximately constant so that $\tilde{\mu} \approx \mu Q_m$ and during sleep $Q_m = 0$, this exhibits very similar dynamics to the two process model, a similarity that can be quantified[13]. In the PCR model, H models the time course of somnogenic chemicals such as adenosine. Parameters for the neuronal variables are taken as physiologically plausible values and parameters χ and μ are then inferred from behavioural data. We have modelled a reduction in the rise of homeostatic sleep pressure during wake with age as a reduction in μ. For the range of values used, since H is close to linear, a change in μ is indistinguishable from a change that would keep χ during sleep constant and vary χ during wake. From a modeling perspective, it is reasonable that the clearance rate is independent of sleep/wake state and that it is the rate of production of somnogenic chemcials that varies with age.

Social constraints

In order to model social constraints, a seven day schedule has been imposed which requires the model to be awake at a fixed alarm time during week days and to stay awake until at least 18:00h. In order to understand how this is implemented it is helpful to understand the structure underlying the PCR model. Since the neuronal timescales $\tau_{v,m}$ are much faster than the homeostatic timescale χ and the circadian timescale, there is a slow manifold given by $V^s_{v,m}(H,C)$. This slow manifold is a folded surface as shown in Fig. S1a. In the absence of social constraints, the slowly varying drive D_v results in switching from wake to sleep and sleep to wake on reaching the saddle-node bifurcations on the slow manifold D_v^+ and D_v^- respectively. On days when there are social constraints, the following procedure is followed. The model is integrated until the alarm time. There are then three scenarios

(i) $D_v < D_V^-$. The model is already in the wake state, so integration is continued as normal.

(ii) $D_v^- < D_v < D_v^+$, then D_v is in the hysteretic region where both sleep and wake are possible states. A switch from sleep to wake is therefore made by instantaneously increasing V_m and decreasing V_v. The equations are then integrated from the new initial conditions.

(iii) $D_v > D_v^+$, then no wake state exists. However, a 'forced' wake state can be reached by increasing the drive to the wake promoting neurons, i.e. increasing D_m, as shown in Fig. S2b. This figure, shows how the position of the hysteretic region depends on the drive to the wake promoting neurons, D_m. The orange curves demark the position of the saddle-node bifurcations as a function of D_m, with D_v^- forming the left hand boundary of the shaded region and D_v^+ the right hand boundary. During spontaneous sleep/wake cycles, D_m is kept fixed and D_v oscillates, tracing out a horizontal line in the D_v, D_m-plane. In the example shown, the alarm goes off when $D_v = 2.64$. At this point, a wake state does not exist, so no change of state can be made by simply making a perturbation to the firing rates. However, increasing D_m as shown by the blue vertical line, moves the system to a point on the edge of the bistable region, enabling a switch to wake to be made. It is this increase of D_m from its default value that constitutes the 'wake effort'. As the day continues, D_v decreases, enabling D_m to decrease back to its normal value while maintaining wake, i.e. moving along the red line. The time spent traversing the red portion of the curve is the time for which wake effort is needed. Specifically, for values of $D_v > D_v^+$ we solve the circadian oscillator equations (4)-(7) along with

$$\chi \frac{dH}{dt} = -H + \mu Q_m(D_m^+, D_v),$$

where $Q_m(D_m^+, D_v)$ is the firing rate of the wake promoting neurons at the saddle-node bifurcation denoting the change from wake to sleep. The value of $Q_m(D_m^+, D_v)$ is given by the solution of

$$\begin{aligned} D_v &= V_v - \nu_{vm} Q_m \\ D_m^+ &= V_m - \nu_{mv} Q_v \\ \frac{\nu_{vm}\nu_{mv}}{\sigma'^2} &= (Q_v - Q_{\max})(Q_m - Q_{\max}), \end{aligned} \tag{13}$$

where

$$Q_{v,m} = \frac{Q_{\max}}{1+\exp[-(V_{v,m}-\theta)/\sigma]}.$$

The forced wake state is continued until $D_v = D_v^+$, and the procedure outlined under (ii) is followed. This is equivalent to the method described in[14] for following the so-called wake 'ghost' during sleep deprivation.

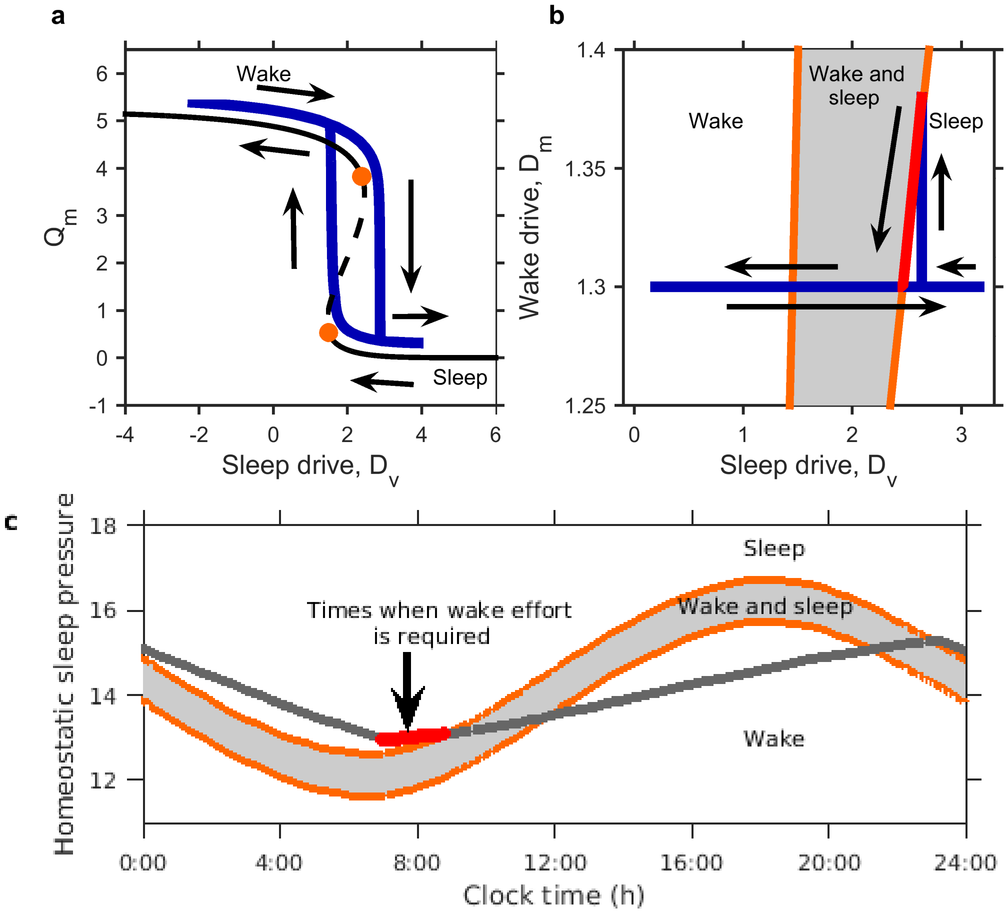

Figure S2. **Dynamics and wake effort**. **a** Slow manifold with a typical sleep/wake trajectory superimposed (offset for clarity). **b** (D_v, D_m)-plane with a typical trajectory for a day where wake effort is required to switch to a wake state. **c** The dynamics re-plotted in two-process model form. The homeostatic sleep pressure (dark grey) decreases during sleep, but is now woken before it reaches the lower threshold. The time for which wake effort is required is marked in red.

If the model switches to sleep during the period after the alarm but before 18:00h, the forced wake regime is again implemented.

The wake effort can also be depicted using the two process model, see Fig.S2c. In terms of the two process model, the region where only the sleep state exists corresponds to the region above the upper threshold. That for which only the wake state exists corresponds to the region below the lower threshold. The region between the two thresholds (shaded grey) is the bistable region where both sleep and wake states exist, $D_v^- < D_v < D_v^+$. Spontaneous wake occurs when the homeostatic sleep pressure reaches the lower threshold, as shown in Fig.S1e. Wake effort is not required if waking occurs sufficiently close to the spontaneous wake time, when the value of the homeostatic sleep pressure lies between the two thresholds. A switch between sleep and wake can then be made by, for example, raising the firing rate of the wake promoting neurons. If waking is required when the homeostatic sleep pressure is still above the upper threshold, where only the sleep state exists, waking cannot occur by a perturbation to the state alone. Applying 'wake effort' by increasing D_m has the effect of shifting the upper threshold upwards, effectively shifting the region for which wake is possible. Wake effort is required until the circadian wake propensity increases sufficiently to counteract the homeostatic sleep pressure. The time for which wake effort is needed is marked in red in Fig.S2c.

Changes across a time zone

It has been shown that the timing of sleep varies across a time zone[15], suggesting that the timing of sleep is not entirely determined by social constraints, but also depends on solar time. We note that a shift of 15° to the West within a single time zone results in a one hour delay of solar noon relative to clock time. The simulations we have presented in Figs. 2-8 set solar noon at a clock time of 12:00h. If solar noon occurs after 12:00h, as is typical to the West of a time zone, spontaneous wake

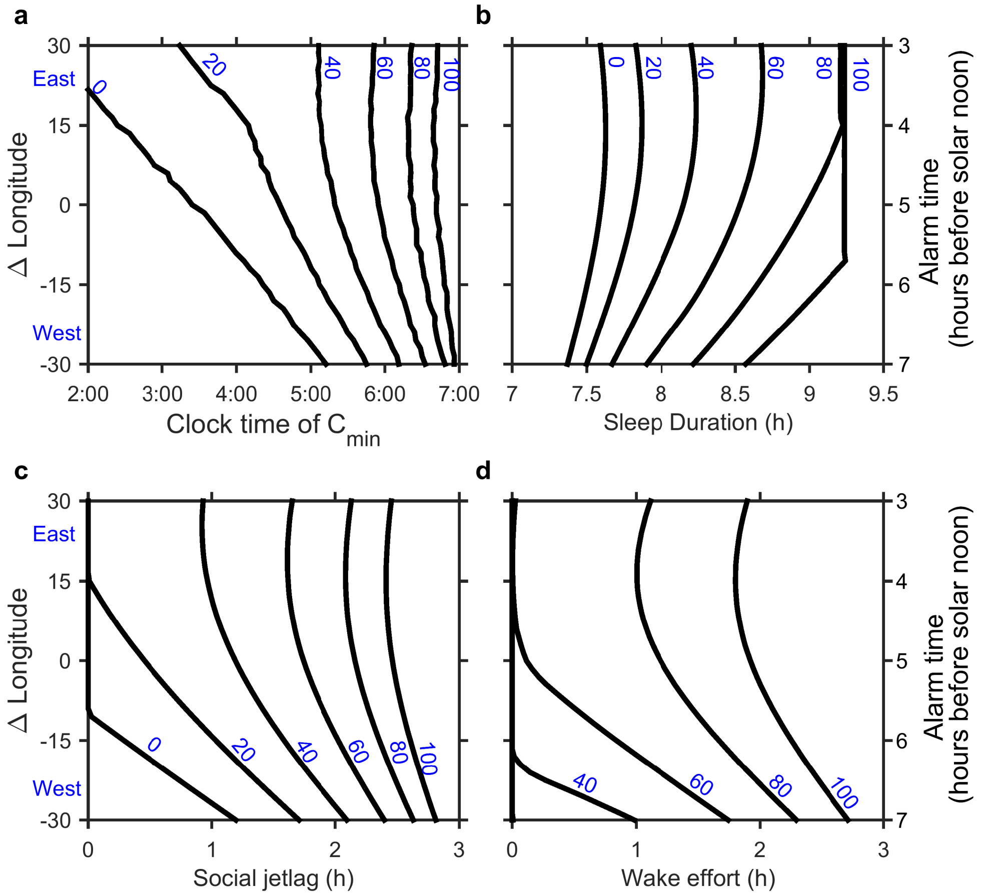

Figure S3. Changes in sleep across a time zone. **a** Minimum of the circadian wake propensity, **b** sleep duration, **c** social jet-lag, **d** wake effort. Results are shown for different levels of evening light. Day light 700 lux. Circadian period 24.2h and the parameters for the homeostatic rise during wake and circadian amplitude are set for age 17y.

will occur at a later clock time than our results suggest. Similarly, if solar noon occurs before 12:00, as is typical to the East of a time zone, spontaneous wake time will occur at an earlier clock time. Since it is the mis-match between the time we are required to wake to satisfy social constraints and our spontaneous wake time that results in social jetlag, we would therefore expect those to the West of a time zone to suffer more than those to the East. Considering the effect of changes in sleep timing due to daylight saving and changes in sleep timing due to changes in social schedules such as school/work start times all come down to the same issue. In all cases, it is the change in alarm clock time versus solar time that is relevant. In Fig. S3 the timing of the circadian minimum, sleep duration, wake effort and social jetlag are all shown for different longitudes. As is expected, these show that those to the West have a later circadian phase, and consequently suffer more from social jetlag, have shorter average sleep durations and require wake effort for longer. We note that although a one hour change in clock time is equivalent to a 15° change in longitude, in practice the irregular shape of time zones mean that many time zones span more than 15°. For example, China, spans more than 60° of longitude yet (excluding Hong Kong) comes under a single time zone, China Standard Time. Some time zones in Russia, Canada, Greenland and South America span more than 30°.

References

1. Phillips, A., Chen, P. & Robinson, P. Probing the mechanisms of chronotype using quantitative modeling. *J. Biol. Rhythms* **25**, 217–227 (2010).
2. The Mathworks, Inc., Natick, Massachussets. *MATLAB version (2015a)* (2015).
3. Phillips, A. & Robinson, P. A quantitative model of sleep-wake dynamics based on the physiology of the brainstem ascending arousal system. *J. Biol. Rhythms* **22**, 167–179 (2007).

4. Forger, D., Jewett, M. & Kronauer, R. A simpler model of the human circadian pacemaker. *J. Biol. Rhythms* **14**, 533–537 (1999).

5. Kronauer, R., Forger, D. & Jewett, M. Quantifying human circadian pacemaker response to brief, extended and repeated light stimuli over the phototopic range. *J. Biol. Rhythms* **14**, 501–515 (1999).

6. Thorne, H., Jones, K., Peters, S., Archer, S. & Dijk, D.-J. Daily and seasonal variation in the spectral composition of light exposure in humans. *Chronobiology Int.* **26**, 854–866 (2009).

7. Wright, K. *et al.* Entrainment of the human circadian clock to the natural light-dark cycle. *Current Biology* **23**, 1554–1558 (2013).

8. Roenneberg, T. *et al.* A marker for the end of adolescence. *Curr. Biol.* **14**, R1038–R1039 (2004).

9. Roenneberg, T. The human sleep project. *Nature* **498**, 427–428 (2013).

10. Dijk, D.-J., Groeger, J., Stanley, N. & Deacon, S. Age-related reduction in daytime sleep propensity and nocturnal slow wave sleep. *Sleep* **33**, 211–223 (2010).

11. Brown, S., Pagani, L., Cajochen, C. & Eckert, A. Systemic and cellular reflections on ageing and the circadian oscillator: a mini-review. *Gerontology* **57**, 427–434 (2011).

12. Skeldon, A., Derks, G. & Dijk, D.-J. Modelling changes in sleep timing and duration across the lifespan: changes in circadian rhythmicity or sleep homeostasis? *Sleep Medicine Reviews* **28**, 92–103 (2016).

13. Skeldon, A., Dijk, D.-J. & Derks, G. Mathematical models for sleep-wake dynamics: comparison of the two-process model and a mutual inhibition neuronal model. *PLoS ONE* **10**, e103877 (2014).

14. Fulcher, B., Phillips, A. & Robinson, P. Modeling the impact of impulsive stimuli on sleep-wake dynamics. *Phys. Rev. E. Stat. Nonlin. Soft. Matter Phys.* **78**, 051920 (2008).

15. Roenneberg, T., Kumar, C. & Merrow, M. The human circadian clock entrains to sun time. *Curr. Biol.* **17**, R44–R45 (2007).
